# Reducing contrast dose using virtual monoenergetic imaging for aortic CTA

**DOI:** 10.1002/acm2.12951

**Published:** 2020-07-02

**Authors:** Ryoichi Yoshida, Keisuke Usui, Yasushi Katsunuma, Hiroshi Honda, Koki Hatakeyama

**Affiliations:** ^1^ Department of Radiology Tokai University Oiso Hospital Kanagawa Japan; ^2^ Department of Radiological Technology Faculty of Health Science Department of Radiation Oncology Faculty of Medicine Juntendo University Tokyo Japan; ^3^ Department of Radiology Tokai University Hospital Kanagawa Japan

**Keywords:** computed tomography, dual‐energy CT, three‐dimensional computed tomographic angiography, virtual monoenergetic imaging, 87.57.Ce, 87.57.Q‐, 87.59.Fm

## Abstract

Three‐dimensional computed tomographic angiography (3D‐CTA) is widely used to evaluate the inner diameters of vessels and the anatomical vascular structure prior to endoscopic aortic surgery or transcatheter valve implantation. Virtual monoenergetic imaging (VMI) is a new application in dual‐energy CT (DECT). We evaluated the potential for contrast dose reduction in preoperative aortic CTA using VMI. To evaluate performance in terms of image quality and vessel shape, we quantified the contrast‐to‐noise ratio (CNR) and the vessel diameter using a cylinder phantom we developed, and used volume rendering to assess visual quality. All VMI had improved CNR values compared with conventional 120 kVp images at an iodine content of 15 mgI/mL. In each image, a virtual mono‐energy of 40 keV yielded the highest CNR value, and an iodine content of 9 mgI/mL was comparable to that of conventional images with an iodine content of 15 mgI/mL. The circularity indices (CI) of the vascular model at 15, 12, and 9 mgI/mL were similar to those of the reference condition using conventional voltages; however, CI was degraded at iodine contents of 6 and 3 mgI/mL with VMI.

In the case of iodine content of 15 mgI/mL, VMI was superior, with conventional image by visual evaluation. In the cases of iodine contents of 12 and 9 mgI/mL, image quality was judged to be almost the same level when comparing 12 and 9 mgI/mL to conventional images. In the case of 6 and 3 mgI/mL, reference image using conventional technique was superior to that of VMI. We demonstrated in that decreasing contrast iodine content is possible using VMI with an energy of 40 keV for preoperative aortic 3D‐CTA.

## 1 INTRODUCTION

Endovascular aortic aneurysm repair and transcatheter aortic valve implantation have increased in frequency due to the advantages of minimally invasive treatment. In these treatment techniques, three‐dimensional computed tomographic angiography (3D‐CTA) is widely used to evaluate the inner diameters of vessels and the anatomical vascular structure prior to the procedure.^1^, ^2^ This image provides important data for selecting the size of the surgical device. Therefore, accurate delineation of the three‐dimensional vessel structures is important, and increasing the contrast of the aorta region is necessary for reconstruction of high‐quality 3D‐CTA. However, iodine‐based CT contrast medium is associated with a risk of hypersensitivity allergic reactions, thyroid dysfunction, and nephropathy.^3^, ^4^, ^5^, ^6^, ^7^, ^8^, ^9^, ^10^ Use of as low a contrast dose as possible for diagnostic imaging has been recommended for minimizing the risk of contrast media‐induced nephropathy.^11^, ^12^, ^13^, ^14^ Therefore, for clinical applications of 3D‐CTA imaging, a technique for image data acquisition that reduces the amount of contrast medium is required.

In dual‐energy CT, virtual monoenergetic imaging (VMI) can increase soft tissue contrast as well as reducing beam‐hardening and scatter artifacts.^15^ Using a lower level of energy nearer the K‐edge energy of iodine can increase the CT number of contrast material to permit injection of a lower dose of iodine.^16^ Increasing the image contrast also increases the image noise when using low‐energy VMI,^17^ which is reflected in the image quality. Several low‐dose CT techniques have been reported that use the iterative reconstruction algorithm and deep learning, including the convolutional neural network.^18^, ^19^, ^20^ Using these techniques, we may be able to improve the contrast of the aortic image and the quality of 3D‐CTA without additional doses. Low‐dose CT could result in a reduction in quantization noise; however, the image contrast of the contrast material itself cannot be improved. Recently, photon‐counting detectors have been used to provide spectral information to enable iodine quantification using a dual‐source CT system,^21^ allowing for separation of the aortic vessel using iodine quantification. However, energy distortion of the incident photon might occur in multiple neighboring pixels, each at a fraction of the true energy, resulting in the degradation of image quality.^22^ In addition, a new image‐processing algorithm has been recently developed (Dual Energy Monoenergetic Plus, Siemens Healthineers, Forchheim, Germany) that can obtain low‐energy images while optimizing the balance between image contrast and noise.^23^ Kraus et al. investigated the effect of the VMI on the visualization of various intramuscular lesions with portal venous phase contrast enhancement using various image quality indexes quantitatively. With regard to the effectiveness of this technique, the researchers clarified that using a VMI with low keV levels can significantly improve the detection of lesions of benign vs malignant intramuscular entities.^24^ In our study, because increasing the contrast of the contrast medium can be realized using a lower level of VMI, a decrease in the volume of contrast media will be clarified quantitatively. Therefore, we investigated the data acquisition conditions that are required when using VMI to reduce the amount of contrast media used in 3D‐CTA aortic imaging. From these results, we can still acquire adequate image information while not impairing the data required from the preoperative image as well as reduce the side effects caused by the contrast media.

To evaluate VMI performance in terms of image quality and vessel shape, we changed the density of the contrast medium step by step using an oval cylinder phantom developed in our department, and evaluated the energy of the VMI conditions and minimum amount of contrast medium required. In this study, we quantified the CNR and the vessel diameter to determine the visibility of aortic 3D‐CTA using volume rendering.

## 2 MATERIALS AND METHODS

### 2.A Phantom design and setup

**Fig. 1:**
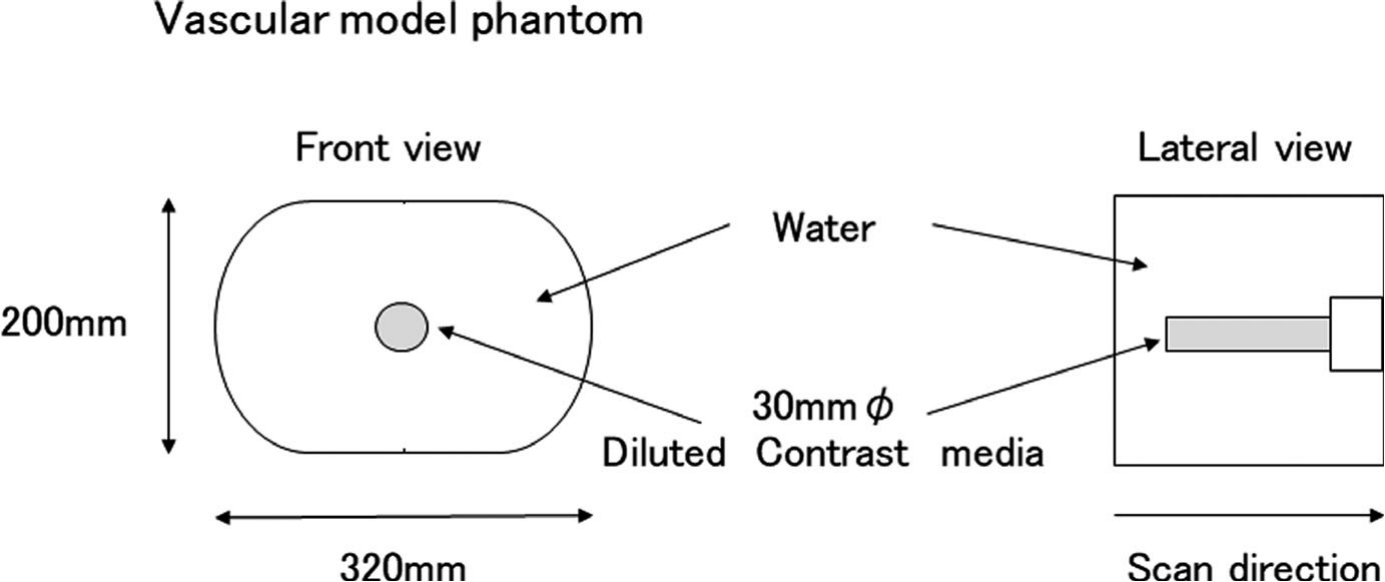
Geometry of the vascular model phantom. The inner lumen was filled with diluted contrast media and fixed in an acrylic elliptical container filled with water. The size of the aortic vascular phantom was 30 mm *ϕ* and was fixed in the center of the container, which had a diameter of 320 × 200 mm.

A vascular model was made with a cylinder of water equivalence, with a diameter of 30 mm to simulate the aorta. For simulating blood concentrations, we deposited dilute iodinated contrast medium solutions containing 3, 6, 9, 12, and 15 mgI/mL. Then, the cylinder was fixed in the center of an acrylic elliptical container (diameter of 320 × 200 mm) filled with water. Figure 1 shows the geometry of the phantom. Image data acquisitions were performed at a volume computed tomography dose index (CTDIvol) of 9.6 mGy with 120 kVp as reference conditions, and modified to be the same CTDIvol in acquiring the dual‐energy CT data by changing the amount of tube current.

### 2.B CT acquisition parameters

All examinations were performed with a third‐generation dual‐source CT scanner (SOMATOM Force, Siemens). The scan parameters were as follows: FOV: 512 × 512 pixels, gantry rotation 0.5 sec, table pitch 0.8, and detector collimation 192 × 0.6 mm. Tube current energies were 70 kVp and 150 kVp with a tin filter in dual‐energy CT data acquisition, and the VMI was reconstructed based on the image data of the two tube voltages using commercial software (Syngo.via ver.20, Siemens).

### 2.C Qualitative analysis

**Fig. 2:**
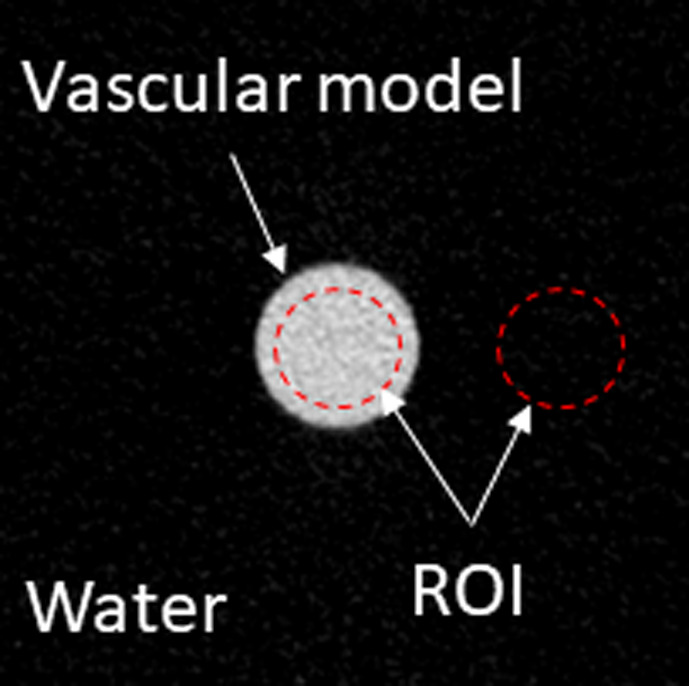
ROI placement onto the reconstructed image in the axial direction. We measured Hounsfield units at the vascular and surrounding water regions. The size of the ROI was 24 mm*ϕ* (shown in the red dashed circle).

The vascular model phantom filled with 15 mgI/mL iodine contrast medium was scanned conventionally for reference data. The vascular model phantoms filled with the five concentrations of iodine were scanned at dual energy. The VMIs were reconstructed in energy range of 40–70 keV. All images were reconstructed into 10 consecutive 1‐mm sections. Regions of interest (ROIs) of diameter 24 mm were drawn within each section and the density measured in Hounsfield units (HU), as shown in Fig. 2. The CNR between the vascular model and the surrounding water on conventional images and each VMIs was calculated for each condition using the formula below:

(1) $$\text{CNR}=\frac{\text{Mean}\;\text{HU}\;\left(\text{vascular}\;\text{region}\right)-\text{Mean}\;\text{HU}\;\left(\text{water}\;\text{region}\right)}{\text{Standerd}\;\text{deviation}\;\left(\text{water}\;\text{region}\right)}$$

To evaluate the circularity, which represents the accuracy of the vascular shape images, we binarized each image by the Otsu method to draw blood vessel shape and calculated the circularity index (CI) using the formula below^25^:

(2) $$\text{CI}=\frac{4\pi A}{P^{2}}$$

where *A* denotes area of the vascular model and *P* denotes perimeter.

### 2.D Visual evaluation

The VMI of the vascular model was compared with the conventional image by visual evaluation. In this study, the VMI was acquired at 40 keV and the conventional image was acquired at 120 kVp. As a reference condition, continuous energy data were scanned for the vascular phantom with the iodine content of 15 mgI/mL, and the VMIs were scanned for each iodine content data (3, 6, 9, 12, and 15 mgI/mL) under same phantom condition. In the visual evaluation, we made a 3D volume rendering (VR) image of the vascular model and evaluated surface irregularities in each iodine image. In the process of this visual evaluation, we adopted the two‐sample preference test suggested by Ferris,^26^, ^27^ and verified not only the superiority of image but also the no difference between images by adding the judgment of neither. In the VR image reconstruction, linear values of HU were converted to the opacity value in the 3D image. The visual evaluation was performed by nine radiological technologists under the same observation conditions.

## 3 RESULTS

### 3.A Qualitative analysis

#### 3.A.1 CNR

**Fig. 3:**
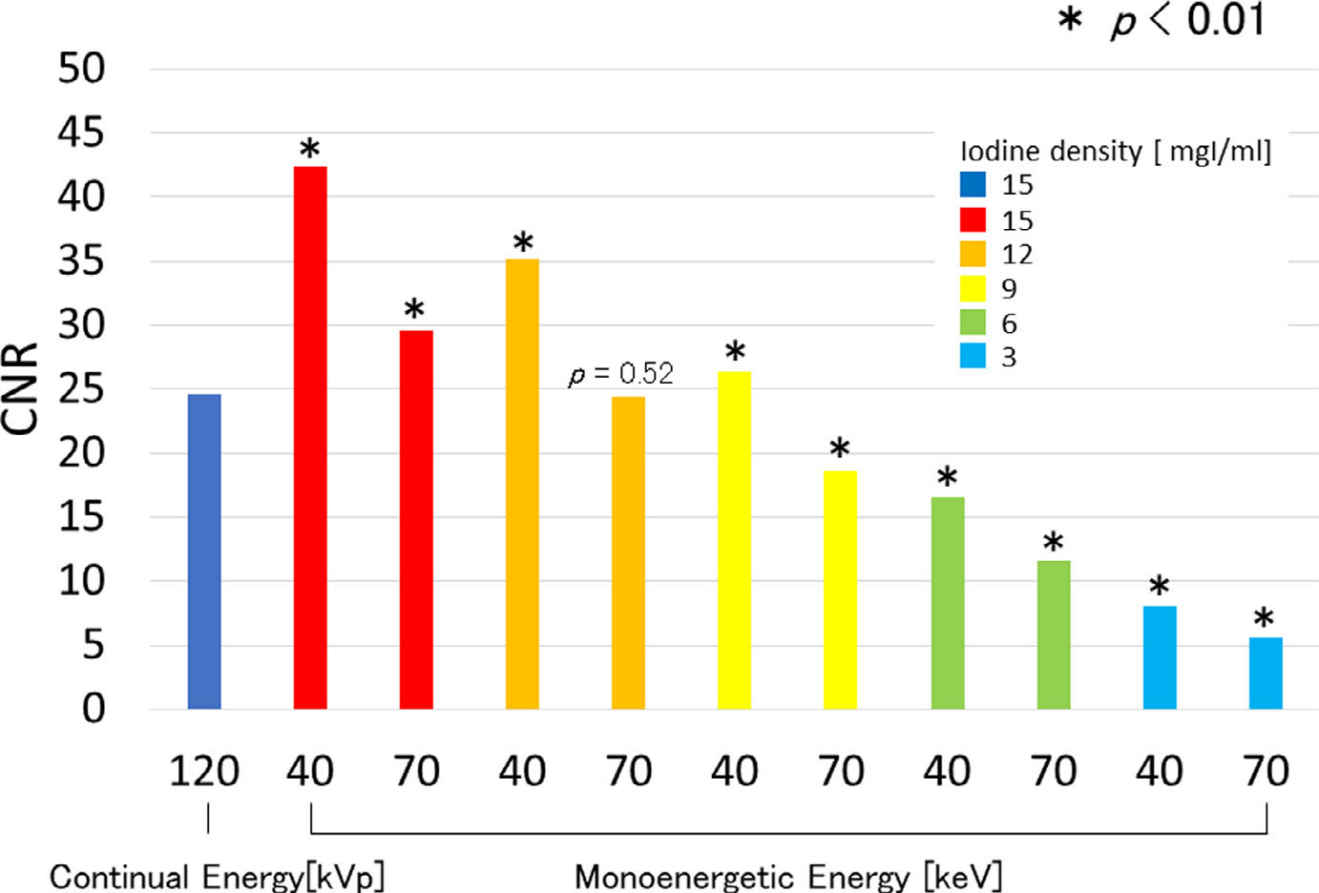
Comparison of CNR between VMIs with different iodine contents with conventional imaging (120 kVp) image at a density of 15 mgI/mL. In the conventional image, continuous energy of 120 kVp was used, and the VMIs were acquired using a monochromatic energy of 40 and 70 keV with contrast medium solutions of 3, 6, 9, 12, and 15 mgI/mL.

Figure 3 shows result of CNR for each iodine content on VMI. In this evaluation, statistical analysis was performed using a two‐tailed t test. Our hypothesis was that there is no difference in CNR between the 120‐kVp image and VMI images. The *P* value was set as 0.01. All images had improved CNR values compared with the conventional images (120 kVp) at the iodine content of 15 mgI/mL. In each iodine content image, the virtual mono‐energy of 40 keV showed the highest CNR value, and the density of 9 mgI/mL was comparable to that of the conventional image acquired with a phantom with iodine content of 15 mgI/mL.

#### 3.A.2 CI

**Fig. 4:**
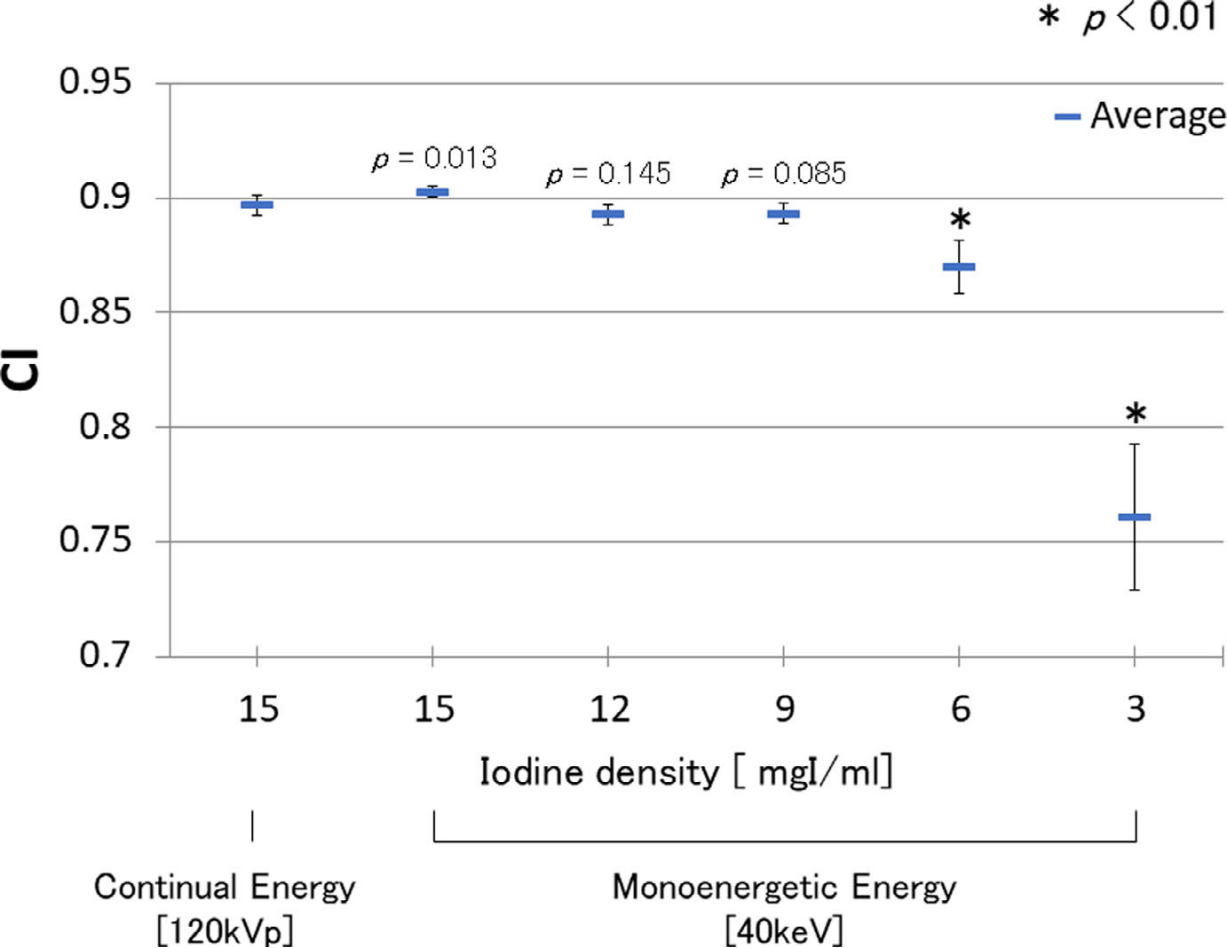
Comparison of CI in the vascular model phantom between VMIs of 40 keV with different iodine contents with the conventional (120 kVp) image with an iodine concentration of 15 mgI/mL. Iodine density was changed to 3, 6, 9, 12, and 15 mgI/mL in the vascular phantom model. The lower and upper margins indicate ± one standard deviation. The mean is marked by the line. We binarized each vascular image using the Otsu method to draw the blood vessel shape and calculated the CI of the different iodine contents.

Figure 4 shows the result of CI in the vascular model phantom. In this evaluation, we performed statistical analysis using a two‐tailed t test. Our hypothesis was that there is no difference in CI between the 120‐kVp image and VMI images. The *P* value was set as 0.01. The CI of the vascular models with iodine contents of 15, 12, and 9 mgI/mL was similar to those of the reference condition using the conventional x‐ray energy; however, CI was degraded in the phantoms with iodine content of 6 and 3 mgI/mL with VMI.

### 3.B Visual evaluation

**Fig. 5:**
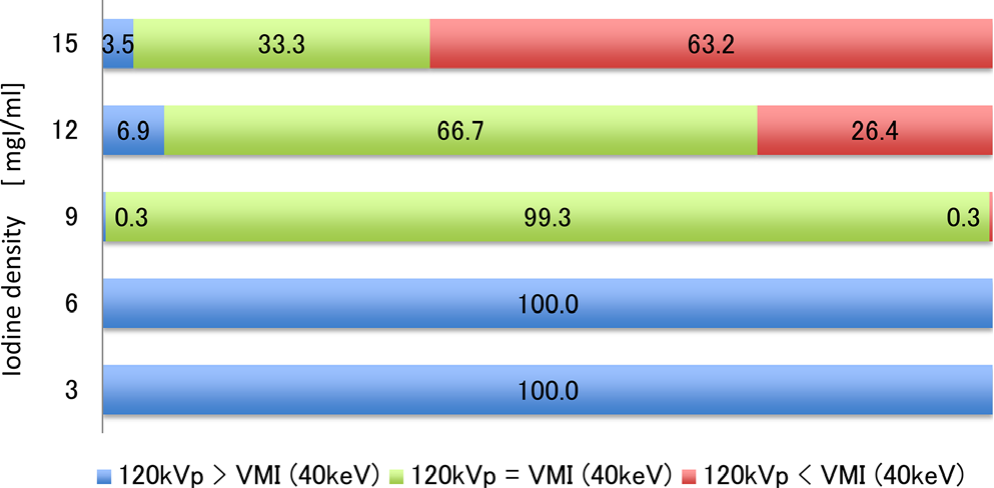
Result of the visual evaluation in the 3D‐VR image. The VMI was acquired at 40 keV, and the conventional image was acquired at 120 kVp. The conventional energy data were scanned with the iodine content of 15 mgI/mL, and the VMIs were scanned with 3, 6, 9, 12, and 15 mgI/mL. The vascular model was compared with the conventional image, and surface irregularities in each VMI with different iodine concentrations were evaluated. The two‐sample preference test was adopted, and we verified not only the superiority of the image but also that there was no difference between images by adding the judgment of neither.

**Fig. 6:**
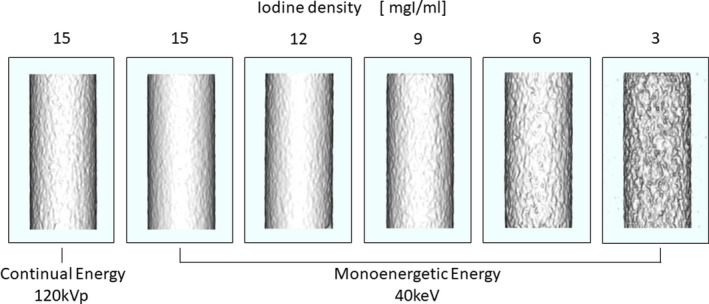
Result of 3D‐VR image of the vascular model with each iodine content. Hounsfield units were linear converted to the opacity value and displayed in direction of the coronal viewing. The VMI was acquired at 40 keV, and the conventional image was acquired at 120 kVp. The iodine content was changed to 3, 6, 9, 12, and 15 mgI/mL. Visual evaluation was performed by nine radiology technologists under the same observation conditions.

Figure 5 shows the results of the visual evaluation, and Fig. 6 shows the 3D VR images for the vascular model of each iodine content as used for the visual evaluation. The results of Fig. 5 show that with an iodine content of 15 mgI/mL, VMI was superior to the conventional image. In cases with an iodine content of 12 and 9 mgI/mL, image quality was judged to be almost the same as that of the conventional images. Moreover, in the case of 6 and 3 mgI/mL iodine content, the conventional reference image was superior to that of VMI.

## 4 DISCUSSION

In this study, we assessed potential reduction in the amount of iodinated contrast media for preoperative aortic 3D‐CTA using VMI and investigated the image quality by qualitative analysis and visual evaluation using a phantom. As shown in Fig. 3, the results of CNR with each density of contrast medium were improved in the VMI with a low energy. This is because image noise was optimized in the process of reconstructing the VMI with low‐energy data, which have been reported in previous studies.^18^ The VMI showed higher CNR values with iodine concentrations of 15 and 12 mgI/mL than the corresponding conventional images. The same CNR values were indicated at iodine concentrations of 9 mgI/mL in VMI and conventional images. The VMI can be created using the energy of 40 keV, as CNR was optimized using the lower energy, as the iodine k‐edge absorption is 33.17 keV. Therefore, VMI can be efficient in improving image contrast. Several studies have evaluated contrast medium reduction focusing on the CNR only. Robert et al. revealed the potential for contrast media reduction of 40%–60% quantifying the CNR data using several commercial CT devices.^16^ We investigated the roundness of the vascular model, and found the CI value was degraded at iodine concentrations < 6 mgI/mL. Therefore, CNR is also an adequate index for evaluating the shape of a blood vessel using the VR image, if we can get sufficient value of CNR, to secure the image quality of the vessel model.

Given the superiority of VMI as a preoperative examination, we visually evaluated the quality of 3D‐VR. In this result, the VMI was better at the same iodine concentrations of 15 and 12 mgI/mL between the VMI and the conventional image. Therefore, the VMI can improve the image quality of 3D‐CTA with reduction of the iodine density by 20% without adding to the radiation dose. This result shows that CNR using VMI with the iodine densities of 15 and 12 mgI/mL was superior than that of CNR by the conventional image. Moreover, with reduction of the iodine content by 40%, the quality of VMI was comparable with the conventional image in the visual evaluation that is shown in Fig. 6. Therefore, compared to conventional images, we can achieve reduction of the iodine concentration by 40% by acquiring 3D‐CTA using VMI with x‐ray energy of 40 keV. From these evaluations, data acquisition conditions using the VMI could be optimized and used to clarify the efficacy of the VMI for selecting the size of the surgical device for the aortic artery. Moreover, the results of this study can help reduce the clinical application of contrast media using the VMI.

Limitations of this study include the need to evaluate the efficacy of VMI in actual human studies. However, our study required multiple rounds of CT data acquisition using several different densities of iodine. Thus, actual human studies are not possible because of the increased risk of patient injury from contrast medium and unnecessary exposure to multiple doses. In this study, the extremely simple structure of the phantom was used to simulate the vascular model. In real patients, this phantom cannot represent the surrounding organs, minute arteries, or veins. In the preoperative imaging of the 3D‐CTA, we must determine the inner diameters of the vessels and the anatomical vascular structure. Therefore, the imaging data are acquired at the most suitable timing focusing the aortic density of iodine, and the diameter of the aorta is measured from the axial image using 3D‐CTA data. Therefore, information about the surrounding organs and veins is not affected by image quality of the 3D‐CTA, and thus, our simulated phantom was suitable for evaluating the preoperative vascular 3D images. If various sized and diameter of vessel require delineation, more sizes of the blood vessel phantoms are needed to test their adequacy. Moreover, the limited x‐ray energy combination of 70 and 150 kV was used for creating VMI. Other x‐ray energy combinations may be better for the quality of VMI.

## 5 CONCLUSION

Image quality of VMI with contrast media at different iodine concentrations was quantified by the physical and the visual evaluations using the phantom data. We demonstrated that a potential decrease of 40% of iodine concentration is possible using VMI at 40 keV for preoperative aortic 3D‐CTA.

## CONFLICT OF INTEREST

No conflicts of interest.
